# ﻿*Demarchushsui* (Coleoptera, Chrysomelidae, Galerucinae, Alticini), a new species from Taiwan, with notes on immatures and biology

**DOI:** 10.3897/zookeys.1177.97854

**Published:** 2023-08-30

**Authors:** Chi-Feng Lee, Jung-Chan Chen

**Affiliations:** 1 Applied Zoology Division, Taiwan Agricultural Research Institute, Taichung 413, Taiwan Taiwan Agricultural Research Institute Taichung Taiwan; 2 No. 16, Lane 75, Shengli East Road, Pingtung City, Pingtung County 900, Taiwan Unaffiliated Pingtung Taiwan

**Keywords:** Host plant, leaf beetles, leaf miner, Loranthaceae, new species, *Taxillusrhododendricolus*, taxonomy

## Abstract

A new species of the little-known genus *Demarchus* Jacoby was discovered at Pilu, East Taiwan, and is here described as *Demarchushsui***sp. nov**. The larvae and adults utilise showy mistletoes as food plants. Their remarkable biology is described in detail, including egg deposition and leaf mining behaviour. Their biology is compared with that of other members of the genus.

## ﻿Introduction

*Demarchus* Jacoby, 1887 is a little-known flea beetle (Coleoptera, Chrysomelidae, Galerucinae, Alticini) with only three species described. The genus was proposed for *D.pubipennis* Jacoby, 1887 from Sri Lanka. A second species, *D.javanus* Bryant, 1941, was described from Indonesia. The third species, *D.nigriceps* Chen & Wang, 1988, was described from China. Odak et al. (1969) reported that *D.pubipennis* caused considerable damage to pigeon pea, *Cajanuscajan* (Linnaeus) (Fabaceae), in India. However, Mushtaque and Baloch (1979) observed that larval and adult *D.pubipennis* fed on leaves of *Loranthuslongiflorus* Desr. (Loranthaceae) in Pakistan, but not pigeon pea, based on testing. *Loranthuslongiflorus* is a species of showy mistletoes, a common name for members of the plant families Loranthaceae. Many members of both families are hemiparasites (Wikipedia 2023). Jolivet and Hawkeswood (1995) reported that *Demarchus* is the only chrysomelid genus whose members utilise Loranthaceae as a food source. Recently, Reid (2017) recorded that species of *Cadmus* (Cryptocephalinae) fed on a narrow range of families, Fabaceae, Myrtaceae, Loranthaceae, and Sapindaceae. Staines (2011) reported that members of *Sceloenoplamultistriata* Uhmann (Cassidinae, Hispines) feed on *Phoradendron* sp. (Loranthaceae). Note that The Angiosperm Phylogeny Group (2016) placed *Phoradendron* within the Santalaceae.

Although the genus *Demarchus* had been redescribed by Maulik (1926), many diagnostic characters for genera proposed by Konstantinov and Vandenberg (1996) are still missing. Results of the current study include redescription of the genus, description of the new species, its immatures, and their remarkable biology.

## ﻿Materials and methods

Dr. Yu-Feng Hsu, a butterfly taxonomist, discovered numerous chrysomelid leaf-miners on *Taxillusrhododendricolus* (Hayata) S.T. Chiu (Loranthaceae), at Pilushenmu (碧綠神木), east Taiwan, during late August 2020. However, rearing success in the laboratory was minimal, with only one adult reared from larvae. During the following year, many more larvae (~ 50) were brought into the laboratory for rearing. Eight adults were successfully reared from larvae but a further 18 adults were collected during late June 2022. This material was sufficient for a detailed taxonomic study.

For rearing studies, more than 50 larvae (see above) were placed in small glass containers (diameter 142 mm × height 50 mm) with cuttings from their host plants. When mature larvae began searching for pupation sites, they were transferred to smaller plastic containers (diameter 90 mm × height 57 mm) filled with moist soil (~ 80% of container volume).

For taxonomic study, five larvae collected from the type locality (see above), and the abdomens of four adults (two collected from the type locality, see above; two reared from larvae) were soaked in hot 10% KOH solution, followed by washing in distilled water to prepare genitalia for illustrations. Head and legs of larvae, and aedeagus, abdominal ventrites, spermatheca, and gonocoxae of adults were dissected from the abdomens, mounted on slides in glycerine, and studied and drawn using a Leica M165 stereomicroscope. For detailed examinations a Nikon ECLIPSE 50i microscope was used. Length of adults was measured from the anterior margin of the eye to the elytral apex, and width at the greatest width of the elytra.

The terminology for larval stages followed Ruan et al. (2020), and for the adult stage Konstantinov and Vandenberg (1996) and Furth (1988).

Exact label data are cited for all type specimens of described species; a double slash (//) separates different labels and a single slash (/) divides the different rows of data on a label. Other comments and remarks are in square brackets: [p] – preceding data are printed, [h] – preceding data are handwritten, [b] – blue label, [w] – white label, and [r] – red label.

Type and non-type specimens or images of both known species of *Demarchus* were studied for comparison, as follows:

*Demarchuspubipennis*. Holotype ♂ (by monotype, The Natural History Museum, London, UK [BMNH]): “Type / H.T [p, w] (circle label with red border) // 4 12/81 [h, w] // Ceylon. / G. Lewis. / 1910–320. [p, w] // Right [h] Hind leg / mounted / in balsam. / S. Maulik, 1929. [p, w] // Galle. / On coast level. / 27.XI.-4.XII.81 [p, w] // *Demarchus* / *pubipennis* Jac [h] / S. Maulik det. [p, w] // Demarchus / pubipennis / Jac. [h, b] // Examined [h] / K. Prathanan / 2005 [p, w]; 1♀ (BMNH): “Larva feeding on leaves / Loranthus longiflorus [h, w] // Kahuta (in Punjab, Pakistan) / 25.VII.74 [h, w] // C.I.B.C / Lor- 7/74- 11 [h, w] // 2022 [h, w] // C.I.E. COLL. / A. [p] 7351 [h, r] // Pres by / Com Inst Ent / B M. 1973-1 [p, w] // Nr. pubipennis ? [h] / det E.A.J. Duffy, 197[p]4 [h, w] // W. Pakistan [h, w]”; 1♂ (BMNH): “On Loranthus / Aug. 1929 / Peechi (in Kerala, South India) / Nair. K. S. S. [h, w] // Sebae the Baly ? / pubipennis Baly [h, y] // Demarchus / pubipennis Jac. [h] / det. M.L. Cox, 198[p]1 [h, w] // Ch. 1(a) [h, w] // C.I.E. COLL. / A. [p] 13361 [h, y] // Pres By / Com Inst Ent / B.M. 1981-1 [p, w]”1♀ (BMNH): “On Loranthus / Aug. 1929 / Peechi (in Kerala, South India) / Nair. K. S. S. [h, w] // Ch. 1(b) [h, w] // C.I.E. COLL. / A. [p] 13361 [h, y] // Pres By / Comm Inst Ent / B.M. 1981-1 [p, w]” 1♂ (BMNH): “Mus. / Collr. / Calcutta [p] (in West Bengal, India) / 31-X-[h]07 [p, w] // Pres By / Com Inst Ent / B M 195[p]3-597 [h, w]”; 1 (glued on the card, sex undetermined) (BMNH): “Fraserpet, / Corrg. (in India) / F.R.I. Sandal / Insect Survey / 16[p]IV[h]30 [p, w] // 1041 [h, w] // Demarchus [h, w]”; 1♂ (BMNH): “Colombo / Ceylon, Sept. 1923 [h, w] // Feeding on / *Loranthus* sp. [h, w] // Pres By / Com Inst Ent / B M 195[p]3-597 [h, w]”.

*Demarchusjavanus*. 1 (sex undetermined, abdomen lost) (BMNH): “Java. [p, w] // Bowring. / 63.47* [p, w] // ? Demarchus sp. [h] / det. M.L. Cox[p], 2000 [h, w]”.

*Demarchusnigriceps* (based on images). Holotype ♂ (by original designation, Institute of Zoology, Chinese Academy of Sciences, Beijing, China [IZAS]): “西藏 [p] (Xizang) 墨脫 (Medog) / 800 –1000 m [h] / 中國科學院 [p, w] (Chinese Academy of Sciences) // 1983.V.15 [h] / 采集者 (collector): 韩寅恒 (Heng-Yin Han) [p, w] // HOLOTYPE [p, r] // *Demarchus* / *nigriceps* [h] // 鑑定者 (determiner): 陳世驤 (Sicien Chen) [p, w].

### ﻿ Demarchus hsui sp. nov.

Taxon classificationAnimaliaColeopteraChrysomelidae

54EFA1B6-73A9-5EE8-A1E9-962D8A0F495D

https://zoobank.org/6F1961AF-38D3-4C8D-AE84-B2E5127098AB

**Figure 1.**
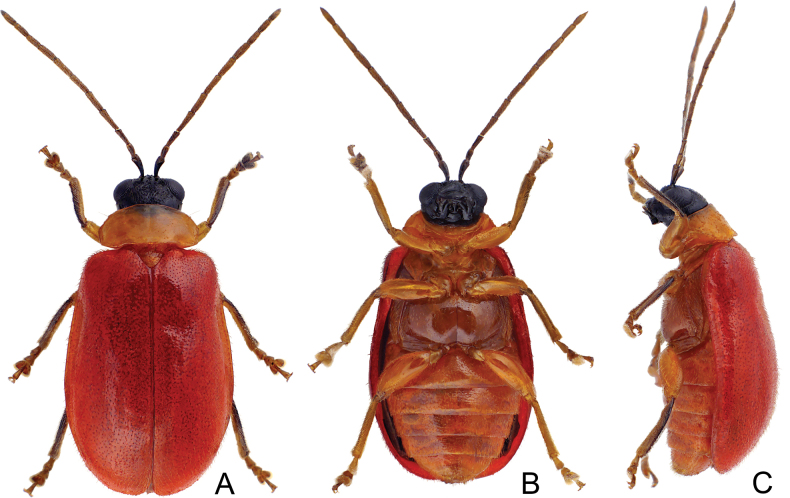
*Demarchushsui* sp. nov. female **A** dorsal view **B** lateral view **C** ventral view.

**Figure 2.**
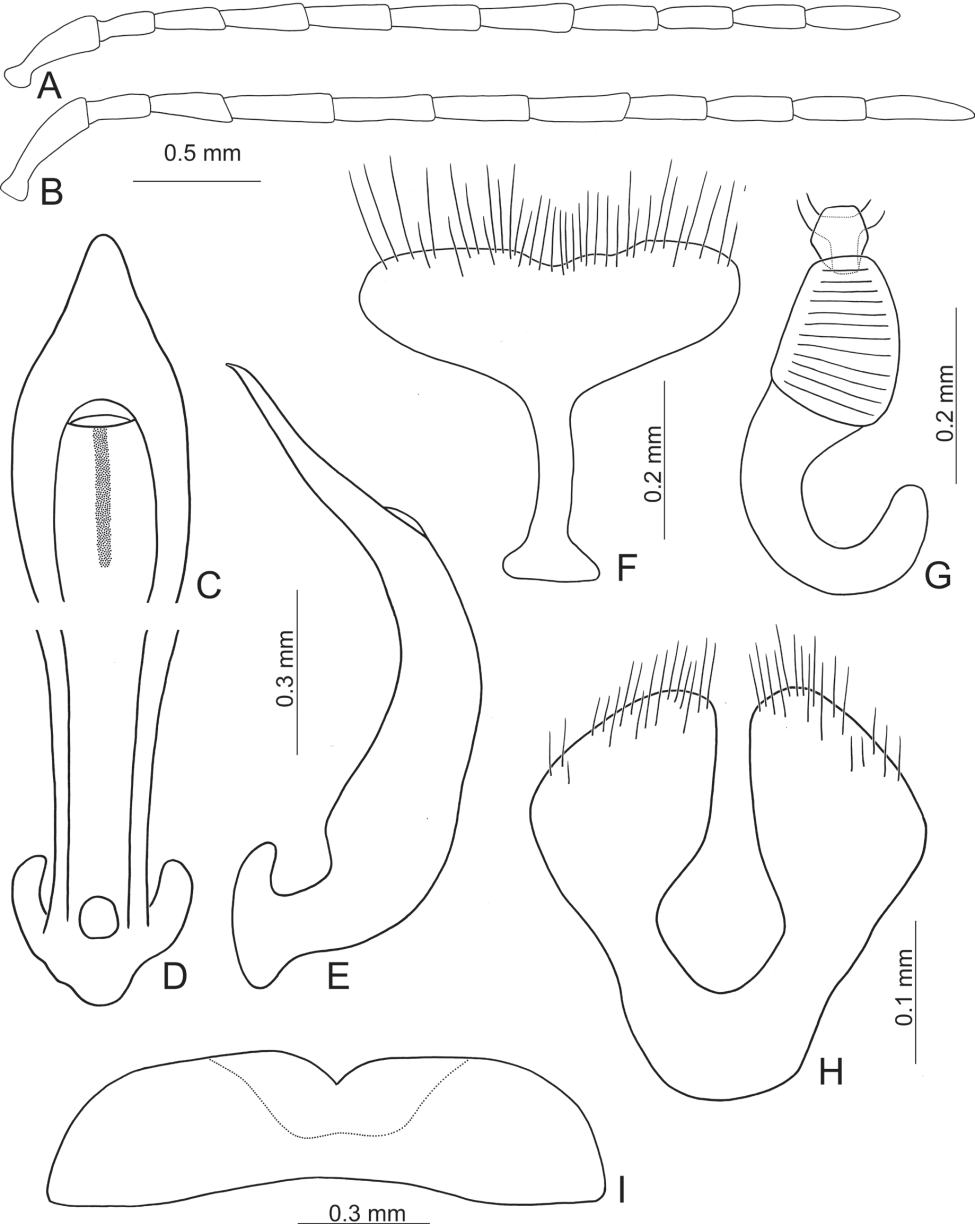
*Demarchushsui* sp. nov. adult **A** antenna, male **B** antenna, female **C** apex of aedeagus, front view **D** base of aedeagus, dorsal view **E** aedeagus, lateral view **F** abdominal ventrite VIII, female **G** spermatheca **H** gonocoxae **I** abdominal ventrite V, male.

**Figure 3.**
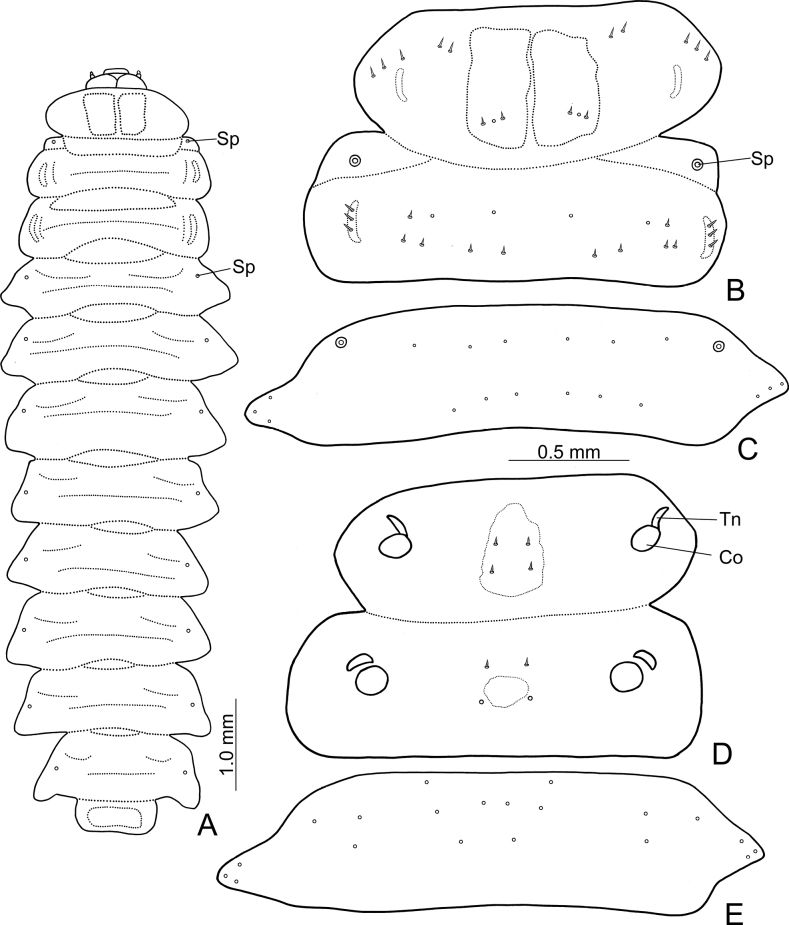
*Demarchushsui* sp. nov. mature larva. **A** dorsal view. **B** pro-mesothorax, dorsal view **C** abdominal segment I, dorsal view **D** pro-mesothorax, ventral view **E** abdominal segment I, ventral view. Abbreviations: Co-Coxa; Sp-spiracle; Tn-Trochantin.

**Figure 4.**
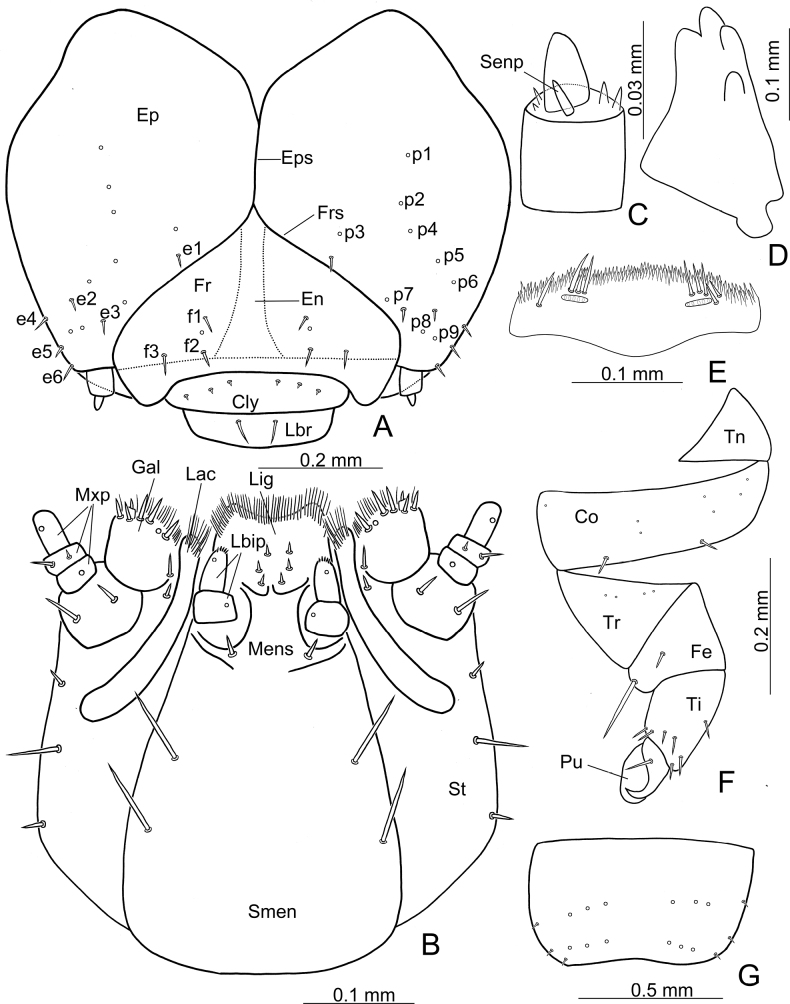
*Demarchushsui* sp. nov. mature larva **A** head **B** maxilla and labium **C** antenna **D** mandible **E** epipharynx **F** middle leg **G** abdominal segment IX, dorsal view. Abbreviations: Cly-clypeus; Co-coxa; e1-e6-epicranial setae; En-endocarina; Ep-epicranium; Eps-epicranial suture; f1-f3-frontal setae; Fe-femur; Fr-frons; Frs-frontal suture; Gal-galea; Lac-lacinia; Lbip- labial palpus; Lig-ligula; Mens-mental sclerite; Mxp-maxillary palpus; p1-p9-epicranial pores; Pu-pulvillus; Scl-sclerotisation; Senp-sensory papilla; St-stipes; Ti-tibia; Tn-trochantin; Tr-trochanter.

**Figure 5.**
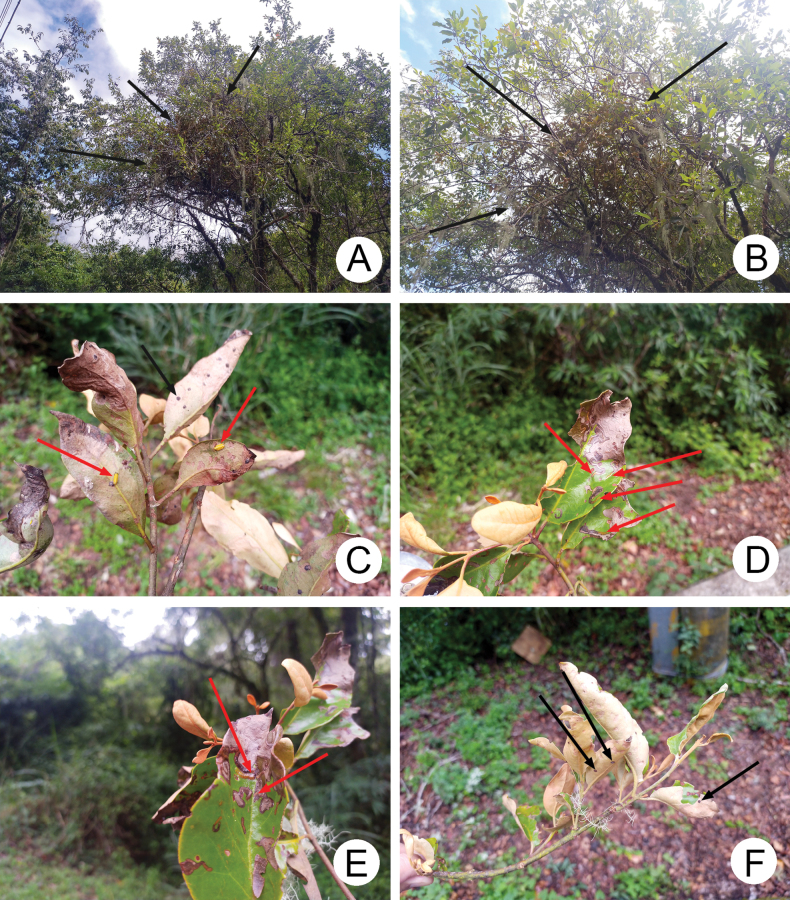
Field photographs taken from the type locality, Pilu (碧綠) **A** host plant, *Taxillusrhododendricolus* (indicated by arrows) **B** close-up and another angle of *T.rhododendricolus***C** branch of *T.rhododendricolus* with egg masses (indicated by black arrows) and larvae (indicated by red arrows) **D** branch of *T.rhododendricolus* with young larvae (indicated by arrows) mining leaves **E** branch of *T.rhododendricolus* with older and younger larvae (indicated by red arrows) mining leaves **F** branch of *T.rhododendricolus* with egg masses (indicated by black arrows).

**Figure 6.**
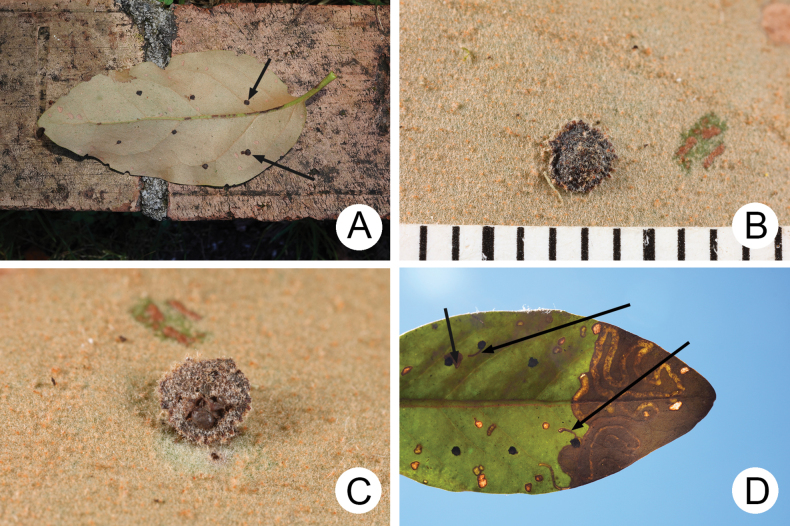
Egg masses of *Demarchushsui* sp. nov. **A** typical distribution of egg masses of *Demarchushsui* sp. nov. on underside of leaf **B** egg mass removed from point where it was deposited, scratch marks indicated by arrows **C** egg mass from a different angle with eggs exposed (indicated by arrow) **D** backlit image with tunnels constructed by the new hatched larvae indicated by arrows.

**Figure 7.**
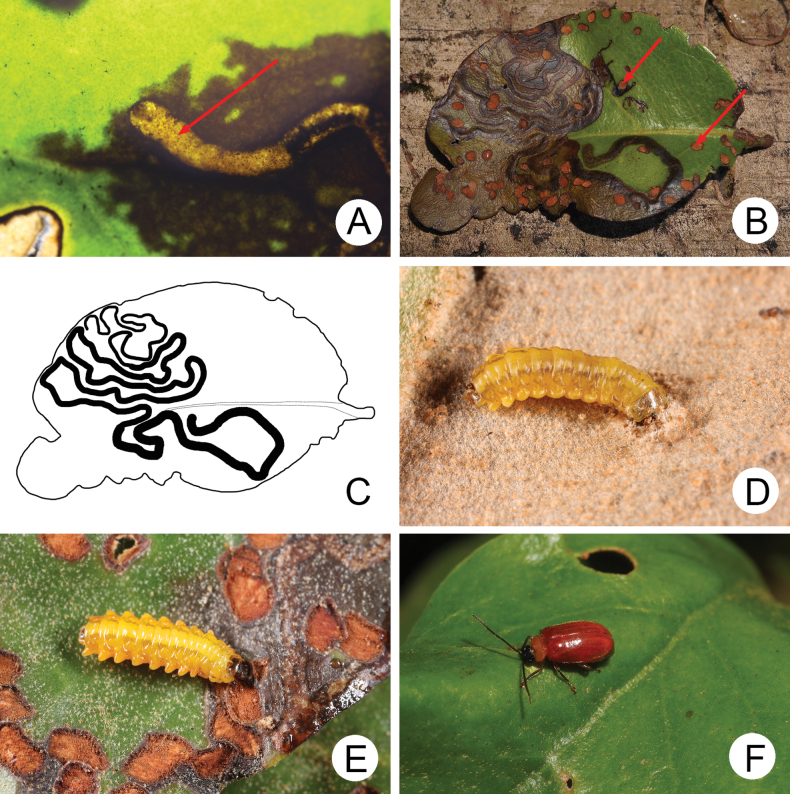
Larvae and adult of *Demarchushsui* sp. nov. **A** young larva (indicated by arrow) mining leaf **B** larval tunnels and feeding marks made by adults on leaf (indicated by arrows) **C** diagrammatic illustration of larval tunnels for Fig. 9B**D** older larva starting to mine leaf **E** mature larva that emerged from larval tunnel **F** adult feeding on leaf.

Figs 1 , 2 , 3 , 4 , 5 , 6 , 7

#### Type material.

***Holotype*** ♂ (TARI, The Insect Collection, Applied Zoology Division, Taiwan Agricultural Research Institute, Taichung, Taiwan): Taiwan. Hualien: Pilu (碧綠), 20.VI.2022, leg. Y.-F. Hsu. Paratypes: 7♂, 10♀ (3♂, 3♀: BMNH; 4♂, 7♀: TARI), data same as holotype; 4♂, 4♀ (TARI) same locality as holotype, 13.VII.2022, leg. Z.-I. Chen.

#### Additional material examined.

Five mature larvae (TARI), same locality as holotype, 20.IX.2022, leg. Y.-F. Hsu.

#### Description.

**Adults.** Colour (Fig. 1A–C) reddish brown, head black, but antenna dark brown or black, prothorax pale yellow, legs yellow with outer margins blackish brown. Pronotum transverse, 2.0× wider than long, disc convex and with lateral fovea, disc with sparse, coarse punctures, lacking antebasal transverse groove; lateral margin rounded, anterior margin slightly concave, posterior margin slightly convex. Elytra slightly wider posteriorly, with shallow transverse impression, widest at apical 1/3, apex convergently rounded, 1.5–1.7× longer than wide, disc with dense, fine punctures and dense pubescence.

**Male.** Length 4.8–5.5 mm, width 2.2–2.5 mm. Antenna filiform (Fig. 2A), ratio of length of antennomeres I to XI 1.0: 0.5: 0.6: 0.8: 0.8: 0.9: 0.9: 0.8: 0.7: 0.7: 0.9; ratio of length to width of antennomeres I to XI 3.0: 2.4: 3.1: 3.0: 3.2: 3.3: 3.4: 3.6: 3.2: 3.2: 3.9. Aedeagus (Fig. 2C–E) with apical 1/2 lanceolate, apex narrowly rounded, basally narrowed; strongly curved in lateral view, slightly recurved near base; tectum slightly sclerotised, with median, longitudinal, strongly sclerotised area from basal margin; endophallic sclerites absent. Apex of abdominal ventrite V (Fig. 2I) with median, angular notch, internally covered by flattened sclerite.

**Female.** Length 5.1–6.0 mm, width 2.4–3.0 mm. Antenna (Fig. 2B) similar to males, ratio of length of antennomeres I to XI 1.0: 0.5: 0.6: 0.9: 0.9: 0.8: 0.8: 0.7: 0.7: 0.6: 0.9; ratio of length to width of antennomeres III to XI 3.6: 2.6: 2.7: 3.7: 3.6: 3.5: 3.7: 3.4: 3.5: 3.0: 4.5. Ventrite VIII (Fig. 2F) weakly sclerotised, T-shaped, with dense, short setae along apical margin, apical margin irregular, spiculum short. Spermathecal receptaculum (Fig. 2G) slightly swollen; pump long and strongly curved, apex widely rounded; spermathecal duct short, shallowly projecting into receptaculum. Gonocoxae (Fig. 2H) short and widely conjoined at base, each gonocoxa widest at apical 1/3, with dense setae along apical areas.

#### Diagnosis.

Adults of this new species are similar to those of *D.nigriceps* in colour pattern, but differ in possessing black antennae and outer margins of tibiae (Fig. 1A–C) (yellow antennae and tibiae in *D.nigriceps* (Fig. 11C, F)), pronotum without antebasal transverse groove (Fig. 1A) (pronotum with antebasal transverse groove in *D.nigriceps* (Fig. 11C)), elytra with transverse impression (Fig. 1A) (elytra without transverse impression in *D.nigriceps* (Fig. 11C)), antennomeres IV-VII subequal in length and longer than antennomere III (IV-VII subequal in length and shorter than antennomere III in *D.nigriceps*), antennomeres VIII-X subequal in length and shorter than antennomere XI (antennomere VIII-XI subequal in length in *D.nigriceps*).

#### Mature larvae.

Length 9.5–9.6 mm, width 2.5–2.6 mm. Live specimens (Fig. 7E): body form elongate, flattened; pale yellow, head and legs blackish brown; prothoracic and abdominal tergite IX with large sclerotised patches; thoracic tergites with small, longitudinal, curved sclerotised patches at sides; thoracic ventrites with small rounded sclerotised patches medially; lateral margins of meso- and metathoracic, and abdominal segments I–VIII expanding outwards, abdominal segments I–VIII each bearing one small process at lateral margins. body bearing tiny setae, the latter sometimes reduced to pores. Spiracles present on mesothorax and abdominal segments I-VIII (Fig. 3A).

Head (Fig. 4A). Flattened, narrower than prothorax, partly retracted into prothorax; frontal sutures (Frs) V-shaped, epicranial suture (Eps) short; endocarina (En) wide. Stemmata absent. Epicranium (Ep): with six pairs of short setae (e1–7) and nine pairs of pores (p1–9); e4–6 situated at posterolateral part of epicranial halves. Frons (Fr): with three pairs of short setae (f1–3) and one pair of pores. Clypeus (Cly): transverse, with three pairs of tiny setae near base. Clypeus and frons devided by apistomal sulcus. Labrum (Lbr): transverse, with one pair of short setae near midline; apical edge rounded. Epipharynx (Fig. 4E): densely setose anteriorly; with four or five large setae on each side; sensilla arranged in one pair of transverse rows. Mandibles (Fig. 4D): symmetrical, palmate, each mandible with four sharp teeth, without penicillus. Antennae (Fig. 4C): weakly sclerotised, two segmented, attached to membranous area at end of frontal suture; first antennomere partly membranous, bearing one small conical sensory papilla and several sensilla; second antennomere small, without sensilla. Maxilla (Fig. 4B): Stipes (St) elongate, bearing one pair of long setae and two pairs of short setae near lateral margin; with a long, curved sclerotisation (Scl). Mala with galea (Gal) and lacinia (Lac) not fused; galea wide, bearing six stout setae and numerous hair-like setae at apex; apical part of lacinia with dense hair-like setae; maxillary palpus (Mxp) three-segmented, second palpomere bearing two setae, one and third palpomeres each bearing one sensilla. Labium (Fig. 4B): submentum (Smen) trapezoid, bearing two pairs of long setae at sides; mentum not well defined; prementum short and transparent, with horseshoe-shaped mental sclerite (Mens), bearing one pair of setae at base; ligula (Lig) membranous, not separated from prementum, anterior edge broadly concave, bearing numerous hair-like setae; labial palpi (Lbip) small, two segmented; with three pairs of sensilla near labial palpi.

Thorax. Prothorax: dorsum (Fig. 3B) with one pair of pores and two pairs of short setae at basal areas of sclerotised patches; two pairs of short setae near base halfway between sclerotised patches and bases of lateral process; three pairs of short setae at sides. Sternal region (Fig. 3D) with one small, sclerotised patch medially, two pairs of short setae at anterior and posterior parts of sclerotised patch respectively. Mesothorax: dorsal region (Fig. 3B) with pores and short setae arranged into two transverse rows, anterior row with two pairs of pores and one pair of setae, posterior row with four setae; lateral longitudinal, sclerotised patches bearing three short setae. Sternal region (Fig. 3D) with one very small, sclerotised patch, one pair of short setae and one pair of pores at anterior and posterior parts outside sclerotised patch. Metathorax: same pattern as mesothorax, except for absence of spiracle. Legs (Fig. 4F): five segments; trochantin (Tn) triangular, without setae or pores; coxa (Co) transverse, bearing several pores at basal half, and two short setae near apical margin; trochanter (Tr) triangular, lacking setae but with several pores; femur (Fe) small, with one long seta on mesal margin, and one small setae at inner face; tibia (Ti) enlarged at base decreasing toward apex, bearing seven short setae at apical 1/2; tarsungulus sclerotised, falciform, bearing one basal setae; pulvillus (Pu) bladder-like, as long as tarsungulus.

Abdomen. Segments I-VIII: dorsal region (Fig. 3C) lacking setae, pores arranged into two transverse rows, bearing three pairs of pores at anterior and posterior row respectively, and three pairs of pores on lateral process; sternal region (Fig. 3E) with pores arranged into three transverse rows, one pair of pores in anterior row, four pairs of pores in middle row, and two pairs of pores in posterior row, three pairs of pores on lateral process. Segment IX (Fig. 4G): pygidium moderately sclerotised; disc with pores arranged into two transverse rows, three pairs of pores in anterior and posterior rows respectively; three pairs of short setae along lateral margin.

#### Host plant.

Loranthaceae: *Taxillusrhododendricolus* (Hayata) S.T. Chiu.

#### Biology.

Larvae are leaf miners of *Taxillusrhododendricolus*, which is a hemiparasite. More than 20 larvae (Fig. 5C–E) were collected from branches (Fig. 5A, B) cut from the host tree, SalixfulvopubescensHayatavar.fulvopubescens Hayata (褐毛柳) at a height of approximately six meters during late August 2020. Forest type is mixed coniferous, including *Piceaasperata* Mast., *Tsugachinensis* (Franch.) Pritzel ex Diels., and *Cunninghamiakonishii* Hayata, with some evergreen broad-leaved and deciduous trees. During 2022, 18 adults were collected using sweep nets from the same plant on June 20 by Dr. Hsu (see types). Eight additional adults were collected from the host plant on trees of *Carpinusrankanensis* Hayata on July 13. Some other collecting trips were carried out during different months. These collecting events indicated that adults appear during June and July, egg masses during early August, and larvae only during late August and September, no life stages were found after October, and it is clear that *D.hsui* sp. nov. is an univoltine species. By contrast, populations of *D.pubipennis* in Pakistan are multivoltine, with up to four generations a year (Mushtaque and Baloch 1979).

Egg masses were deposited at some distance from each other on undersides of leaves (Fig. 6A). Females scratched the leaf surface several times (Fig. 6B) so that neonate larvae could burrow into the leaves easily. Then four or five eggs (Fig. 6C) were laid and covered by faeces. Usually only one larva hatched successfully from each egg mass (Fig. 6D) and began mining leaves.

Leaves of *T.rhododendricolus* decayed as soon as larvae constructed tunnels (Fig. 7A). Tunnels made by larvae were always transverse and turned towards the leaf apex (Fig. 7B, C). Larvae turned tunnels basally when conditions were not suitable to maintain the apical direction. Such a feeding pattern caused the entire leaf to decay from apex to base (Figs 5D, E, 6D, 7B). Larvae exited tunnels when conditions deteriorated and searched for more suitable leaves. They were able to tunnel into newly selected leaves and continue development (Fig. 7D). Mature larvae (Fig. 7E) emerged from tunnels and walked or fell to the ground, mainly falling when disturbed. They burrowed into soil and built underground chambers for pupation.

Adults on leaves of *T.rhododendricolus* were active during the day (Fig. 7F). They fed on the upper surface of leaves, leaving round feeding scars (Fig. 7B, E).

#### Remarks.

Larvae of *D.hsui* sp. nov. exhibit unusual characters that are typical for leaf miners (Takizawa 2005), including flattened body and head, head with vertex incised in a U- or V-shape posteriorly, and body surface without setae or tubercles.

#### Etymology.

This new species is named for Dr. Yu-Feng Hsu (徐堉峰), who is a well-known butterfly expert and the first person to collect specimens.

#### Distribution.

The new species is only recorded from the type locality -- Pilu (碧綠), in Hualien County, East Taiwan. It is located at 24°10'51.3"N, 121°24'11.6"E, 2150 m MSL, and protected by the Taroko National Park (太魯閣國家公園). This locality seems to be the biodiversity hotspot. The rarely collected chrysomeline *Ambrostomachinkinyui* Kimoto & Osawa, 1995 is also only known from this locality (Kimoto and Osawa 1995), as well as multiple undescribed species (unpublished data).

### ﻿ Demarchus

Taxon classificationAnimaliaColeopteraChrysomelidae

Jacoby, 1887

B1463FB8-1770-5E54-A83B-93530C30D596

Demarchus Jacoby, 1887: 101 (type species: Demarchuspubipennis Jacoby, 1887, by original designation); Maulik 1926: 135 (redescription); Scherer 1969: 196 (catalogue).

#### Included species.

*Demarchuspubipennis* Jacoby, 1887, *D.javanus* Bryant, 1941, *D.nigriceps* Chen & Wang, 1988, *D.hsui* sp. nov.

#### Redescription.

**Figure 8.**
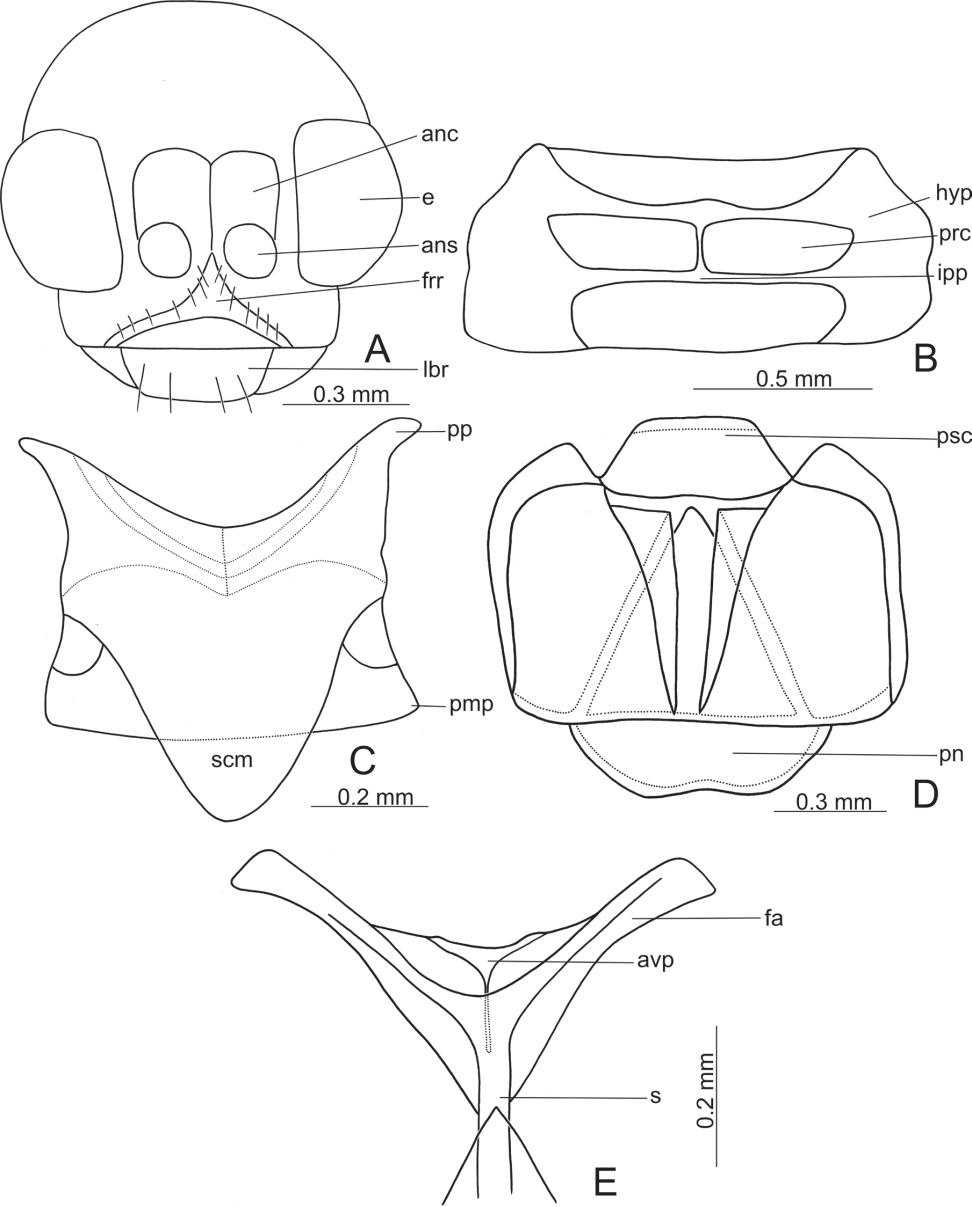
Diagnostic features of adults of *Demarchushsui* sp. nov. **A** head **B** ventral view of prothorax **C** mesonotum **D** metanotum **E** metendosternite. Abbreviations: anc-antennal calli; ans-antennal socket; avp-anterior part of ventral projection; e-eye; fa-furcal arm; frr-frontal ridge; hyp-hypomera; ipp-intercoxal prosternal process; lbr-labrum; pmp-postmedial projection; pn-postnotum; pp-prealar projection; prc-procoxal cavity; psc-prescutum; s-stalk; scm-scutum.

Body elongate rounded, head visible from above. Head (Fig. 8A) drawn into prothorax, hypognathous, broadly oval in frontal view; vertex large, covered with dense, coarse punctures and short setae; antennal calli rectangular, well separated from vertex by deep furrow, not separated from antennal sockets; antennal sockets large, distance between sockets smaller than diameter of socket, sockets separated by frontal ridge, not separated from eyes; frontal ridge triangular, anterior surface of frons convex, bearing short setae at the sides of frontal ridge; frontal area, including mouth region, not separated from genae; eyes small, convex, the longest diameter of eye smaller than the distance between eyes, not delineated by sulci from vertex and frons. Antenna (Fig. 2A, B) 11-segmented, filiform, long, extending beyond middle of body; antennomere I shorter than two following antennomeres combined. Labrum with two pairs of setae.

***Prothorax*.** Pronotum distinctly wider than long, disc glabrous, with antebasal transverse impression, limited laterally by short longitudinal furrows; hypomera (hyp) (Fig. 8B) large, hypomeral sutures reduced; prosternum above procoxal cavities narrow, narrower than width of procoxal cavities, intercoxal prosternal process (ipp) narrow, its anterior edge straight; procoxal cavities (prc) closed, transversely elongate.

***Mesothorax*.** Mesonotum (Fig. 8C) of typical shape, lightly sclerotised, prealar projection (pp) well developed, elongate; postmedial projections (pmp) reduced; scutum (scm) widely rounded. Mesoventrite short, mesanepimera and mesaepisterna narrow.

***Metathorax*.** Metanotum (Fig. 8D) well sclerotised, well developed, and typical for alticines; prescutum (psc) and postnotum (pn) wide. Metaventrite as wide as first abdominal segment, metaventral process reduced, posterior edge of metaventrite medially with deep incision, metaepisterna of typical shape, narrow. Metendosternite (Fig. 8E) with branches of anterior part of ventral process (avp) well developed, short; furcal arm (fa) narrow and well sclerotised; stalk (s) wide and short.

**Figure 9.**
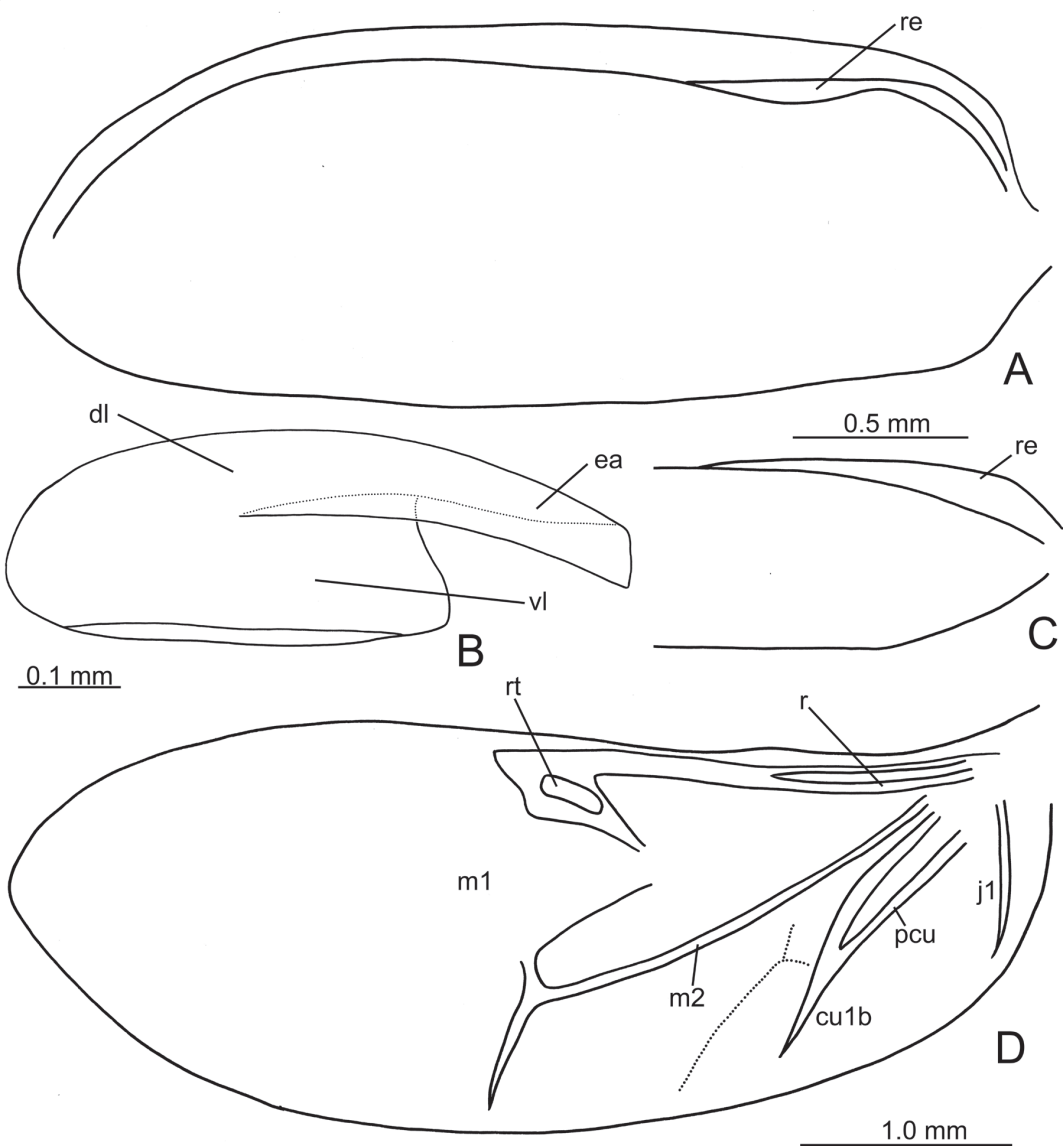
Diagnostic features of adults of *Demarchushsui* sp. nov. **A** elytron, ventral view **B** metafemora spring **C** base of elytra, lateral view **D** hind wing. Abbreviations: cu-cubital vein; dl-dorsal lobe; ea-extended arm; j-jugal vein; m-medial vein; r-radial vein; re-recurved part of epipleuron; rt-sector of radial vein; vl-ventral lobe.

***Elytra elongate oval*.** Humeral callus well developed. Elytral punctures and pubescence dense and confused. Epipleuron (Fig. 9A, B) wide, horizontal, and recurved at apical 1/3, and then vertical, almost reaching elytral apex. Elytral binding patch covered with numerous teeth that are rounded in shape, ventral surface of elytra glabrous.

***Hind wings*.** Wing venation (Fig. 9C) typical for alticines (Konstantinov and Vanderberg 1996), with completely developed wings and no tendency to reduction. Typical set of veins is present; radius (r), sector of radial vein (rt), medial veins 1 (m1) and 2 (m2), cubital vein 1b (cu1b), and precubital vein (pcu). In addition, first jugal vein (j1) is also visible.

***Abdomen***. Ventrites short, wide, without projections or convexities, ventrite I shorter than metasternum; sexual dimorphism present in the shape of ventrite V (apical margin with median notch in males but absent in females); pygidium without medial longitudinal groove; tergite VIII well-developed.

**Figure 10.**
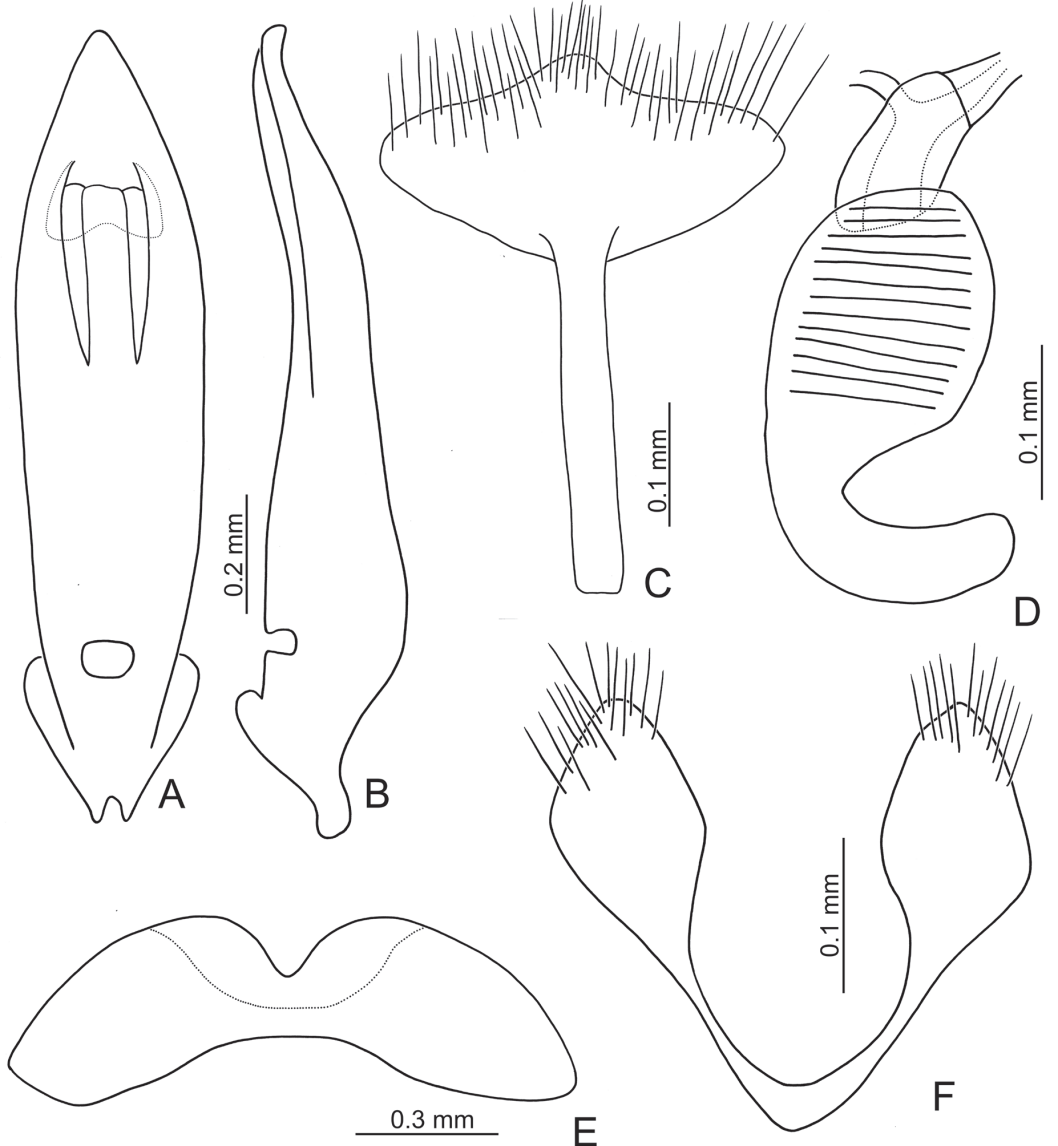
*Demarchuspubipennis* Jacoby, adult **A** aedeagus, dorsal view **B** aedeagus, lateral view **C** abdominal ventrite VIII, female **D** spermatheca **E** abdominal ventrite V, male **F** gonocoxae.

***Male genitalia*** (Figs 2C–E, 10A, B) consisting of median lobe of aedeagus, and Y-shaped tegmen. Aedeagus lacking endophallic spiculae.

***Female genitalia*** consisting of ventrite VIII, gonocoxae, and spermatheca. Ventrite VIII (Figs 2F, 10C) T-shaped, base well-sclerotised, speculum short, slightly longer than wide, its apical margin with dense setae. Spermathecal receptacle (Figs 2G, 10D) slightly swollen, sclerotised spermathecal duct very short, pump long, and strongly curved. Gonocoxae (Figs 2H, 10F) short and wide, basally joined, with dense setae at apical areas.

***Legs*.** Anterior and middle legs of typical shape, without modifications; tibiae without apical spurs, furrows, grooves, ridges, or excavations. Posterior femora slightly swollen; posterior tibiae comparatively short, not longer than length of femora; metafemoral spring simplified (Fig. 9C), lightly sclerotised, dorsal edge of dorsal lobe (dl) flat, extended arm (ea) of dorsal lobe relative long, ventral lobe (vl) cylindrical, apically rounded, without lower part curving dorsally, and no basal angle, ventral edge of ventral lobe recurved; posterior tarsus attached to tibia apically; tarsus slightly longer than half of tibia; metatarsomere I shorter than three following tarsomeres combined, ventrally with short, dense setae. Tarsomeres III bilobed; tarsal claws bifid.

#### Remarks.

One character misjudged by Jacoby (1887) and Maulik (1926) is the closed procoxal cavities. Since the posterior margins of the procoxal cavities are so slender, both authors regarded it as the open. In fact, the posterior margins of the procoxal cavities are not reduced in the type specimens of *D.hsui* sp. nov. and the holotype of *D.pubipennis*.

*Demarchus* is easily recognised by the following combination of characters: pubescent elytra, glabrous pronotum, closed procoxal cavities, and unique shape of elytral epipleura, typical form of the Pyrrhalta-like Morpho-Group which, was defined by Furth and Suzuki (1998) based on the metafemoral spring.

#### Biology.

Immature stages and biology for *Demarchuspubipennis*, reported by Mushtaque and Baloch (1979), occur on Loranthaceae and larvae are leaf miners. Assertions by Odak et al. (1969) are not supported because the host plant, *Cajanuscajan* L., belongs to the Fabaceae. Larvae and adults of *D.pubipennis* did not feed on this plant when tested by Mushtaque and Baloch (1979). Moreover, Odak et al. (1969) indicated that the larvae were root feeders, which is incorrect since they possess morphological characters that are typical of leaf miners, and this lifestyle has been confirmed through field and laboratory observations. The current study confirms that adults of *D.hsui* sp. nov. feed on leaves of another species of Loranthaceae, *Taxillusrhododendricolus*, and their larvae are also leaf miners.

**Figure 11.**
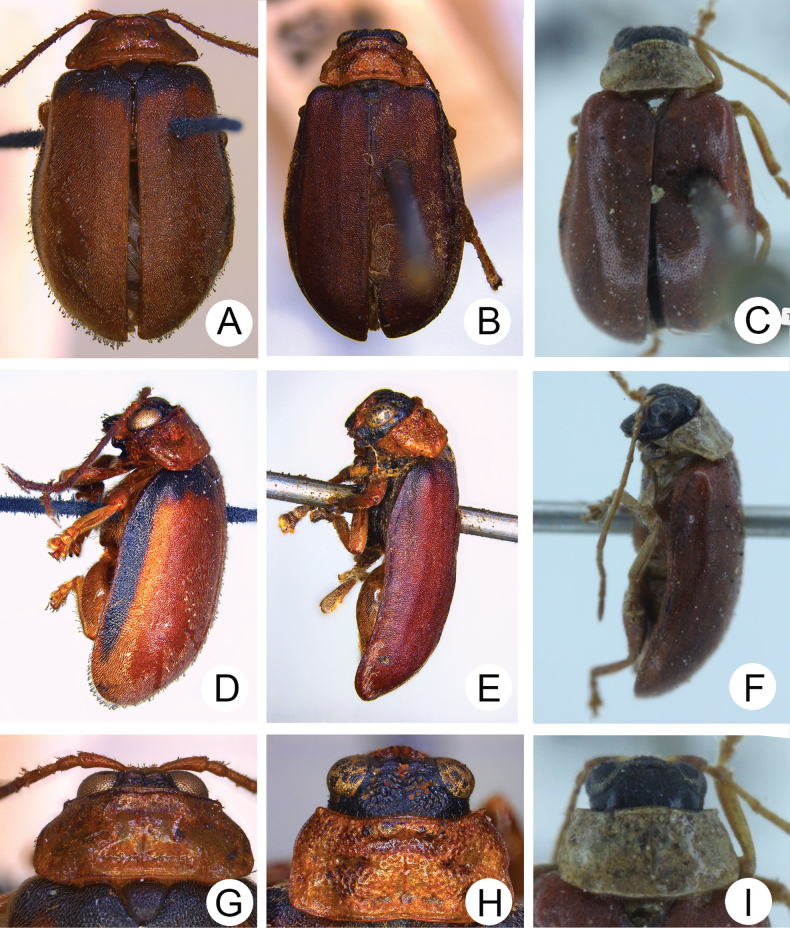
Diagnostic characters of *Demarchuspubipennis* Jacoby, non-type male from Sri Lanka; *D.javanus* Bryant, non-type adult from Java; *D.nigriceps* Chen & Wang, holotype. Dorsal view: **A***D.pubipennis***B***D.javanus***C***D.nigriceps*; lateral view: **D***D.pubipennis***E***D.javanus***F***D.nigriceps*; head and pronotum: **G***D.pubipennis***H***D.javanus***I***D.nigriceps*.

#### Distribution.

Sri Lanka, India, Pakistan, China (Xizang), Indonesia (Java), Taiwan.

### ﻿Key to adults of *Demarchus* species of the world

**Table:** 

1	Elytra yellowish brown or dark brown, with basal areas darker (Fig. 11A, B); discs of elytra with extremely dense punctures and pubescence (Fig. 11A, B, D, E)	**2**
–	Elytra entirely reddish brown (Figs 1A, 11C); discs of elytra with dense punctures and pubescence (Figs 1A, C; 11C, E)	**3**
2	Elytra widely rounded (Fig. 11A), dark areas on basal margin extending into middle of lateral margins (Fig. 11D); punctures on pronotum medially absent (Fig. 11G)	***D.pubipennis* Jacoby**
–	Elytra parallel-sided (Fig. 11B), dark areas on basal margin not extending into lateral margins (Fig. 11E); punctures on pronotum entirely dense (Fig. 11H)	***D.javanus* Bryant**
3	Antennae yellowish brown, tibiae entirely yellow-brown (Fig. 11C, F); pronotum with antebasal transverse groove (Fig. 11 I); elytra without transverse impression (Fig. 11C)	***D.nigriceps* Chen & Wang**
–	Antennae dark brown or blackish brown, outer margins of tibiae yellow (Fig. 1A–C); pronotum without antebasal transverse groove (Fig. 1A); elytra with transverse impression (Fig. 1A)	***D.hsui* sp. nov.**

## Supplementary Material

XML Treatment for Demarchus hsui

XML Treatment for Demarchus

## ﻿Additional information

### Author ORCIDs

Chi-Feng Lee https://orcid.org/0000-0003-1996-0557

### Data availability

All of the data that support the findings of this study are available in the main text or Supplementary Information.
